# Erythrocyte Membrane Fatty Acid Composition as a Potential Biomarker for Depression

**DOI:** 10.1093/ijnp/pyad021

**Published:** 2023-05-22

**Authors:** Ting Liu, Lu Wang, Jimin Guo, Tingyu Zhao, Hui Tang, Fang Dong, Chuanyue Wang, Jindong Chen, Mimi Tang

**Affiliations:** Department of Pharmacy, Xiangya Hospital, Central South University, Changsha, Hunan, China; National Clinical Research Center for Geriatric Disorders, Xiangya Hospital, Central South University, Changsha, Hunan, China; The National Clinical Research Center for Mental Disorders and Beijing Key Laboratory of Mental Disorders and Beijing Institute for Brain Disorders Center of Schizophrenia, Beijing Anding Hospital, Capital Medical University, Beijing, China; National Clinical Research Center for Mental Disorders, China National Technology Institute on Mental Disorders and Department of Psychiatry, The Second Xiangya Hospital of Central South University, Changsha, Hunan, China; Advanced Innovation Center for Human Brain Protection, Capital Medical University, Beijing, China; Department of Microbiology, Immunology and Molecular Genetics, University of California, Los Angeles, Los Angeles, CA, USA; Department of Pharmacy, Xiangya Hospital, Central South University, Changsha, Hunan, China; National Clinical Research Center for Geriatric Disorders, Xiangya Hospital, Central South University, Changsha, Hunan, China; National Clinical Research Center for Mental Disorders, China National Technology Institute on Mental Disorders and Department of Psychiatry, The Second Xiangya Hospital of Central South University, Changsha, Hunan, China; The National Clinical Research Center for Mental Disorders and Beijing Key Laboratory of Mental Disorders and Beijing Institute for Brain Disorders Center of Schizophrenia, Beijing Anding Hospital, Capital Medical University, Beijing, China; Advanced Innovation Center for Human Brain Protection, Capital Medical University, Beijing, China; The National Clinical Research Center for Mental Disorders and Beijing Key Laboratory of Mental Disorders and Beijing Institute for Brain Disorders Center of Schizophrenia, Beijing Anding Hospital, Capital Medical University, Beijing, China; National Clinical Research Center for Mental Disorders, China National Technology Institute on Mental Disorders and Department of Psychiatry, The Second Xiangya Hospital of Central South University, Changsha, Hunan, China; Advanced Innovation Center for Human Brain Protection, Capital Medical University, Beijing, China; National Clinical Research Center for Mental Disorders, China National Technology Institute on Mental Disorders and Department of Psychiatry, The Second Xiangya Hospital of Central South University, Changsha, Hunan, China; Department of Pharmacy, Xiangya Hospital, Central South University, Changsha, Hunan, China; National Clinical Research Center for Geriatric Disorders, Xiangya Hospital, Central South University, Changsha, Hunan, China

**Keywords:** Major depression disorder, erythrocyte membrane fatty acids, biomarkers

## Abstract

**Background:**

Major depressive disorders is a chronic and severe psychiatric disorder with poor prognosis and quality of life. Abnormal erythrocyte fatty acid (FA) composition in depressed patients were found in our previous study, but the relationship between erythrocyte membrane FA levels and different severity of depressive and anxiety symptoms remains to be explored.

**Methods:**

This cross-sectional study included 139 patients with first-diagnosed, drug-naïve depression and 55 healthy controls whose erythrocyte FA composition was analyzed. Patients with depression were divided into severe depression and mild to moderate depression or depression with severe anxiety and mild to moderate anxiety. Then the differences of FA levels among different groups were analyzed. Finally, the receiver operating characteristic curve analysis was applied to identify potential biomarkers in distinguishing the severity of depressive symptoms.

**Results:**

Levels of erythrocyte membrane FAs were elevated among patients with severe depression compared with healthy controls or patients with mild to moderate depression of almost all kinds. While C18:1n9t (elaidic acid), C20:3n6 (eicosatrienoic acid), C20:4n6 (arachidonic acid), C22:5n3 (docosapentaenoic acid), total fatty acids (FAs), and total monounsaturated FAs were elevated in patients with severe anxiety compared with patients with mild to moderate anxiety. Furthermore, the level of arachidonic acid, C22:4n6 (docosatetraenoic acid), elaidic acid, and the combination of all 3 were associated with the severity of depressive symptoms.

**Conclusions:**

The results suggested that erythrocyte membrane FA levels have the potential to be the biological indicator of clinical characteristics for depression, such as depressive symptoms and anxiety. In the future, more research is needed to explore the causal association between FA metabolism and depression.

**Significance Statement**

Erythrocyte membrane fatty acids (FAs) could be potential biomarkers, but it still needs to be further investigated. Levels of erythrocyte membrane FA were elevated among patients with severe depression compared with healthy controls or patients with mild to moderate depression of almost all kinds. While C18:1n9t (elaidic acid [EA]), C20:3n6 (eicosatrienoic acid [EET]), C20:4n6 (arachidonic acid [AA]), C22:5n3 (docosapentaenoic acid [DPA]), total FAs, and total monounsaturated FAs were elevated in patients with severe anxiety compared with patients with mild to moderate anxiety. Moreover, the levels of AA, C22:4n6 (docosatetraenoic acid [DTA]), EA, and the combination of all 3 have the ability to distinguish the severity of depressive symptoms. In the future, more research is needed to explore the causal association between FA metabolism and depression as well as the impact of erythrocyte FA metabolism on depression prevention, diagnosis, treatment, and prognosis.

## INTRODUCTION

Major depression disorder (MDD) is a chronic and severe psychiatric disorder with unfavorable prognoses, repeated episodes, and poor quality of life for patients (Daly et al., 2010). The extensively prescribed antidepressants, such as selective serotonin reuptake inhibitors, have demonstrated low response rates, a long onset of efficacy, and significant side effects (Rush et al., 2011). As a result, discovering how to increase the chances of positive therapeutic response or remission while limiting negative side effects is critical for enhancing depression outcomes. Depressive symptoms, such as depressed mood and loss of interest, are common in a variety of mental disorders and hence require special attention. Current editions of the Diagnostic and Statistical Manual of Mental Disorders (DSM) and International Classification of Diseases have made consistent standards for clinical diagnosis and research of mental disorders (Regier et al., 2009; Clark et al., 2017). However, some issues have been raised that neuroscience and genetics findings are not fully consistent with the clinical consensus in the psychiatry (Insel et al., 2010). One of the main goals of the current psychiatry research field is to find biological markers that may be utilized to diagnose diseases or better predict therapy responses.

The monoaminergic theory of MDD has long been the most popular, and most antidepressants are thought to primarily work by modulating monoaminergic neurotransmission. Previous research has also highlighted that inflammatory, epigenetic regulation, the development of brain structures, and stress hormones (cortisol) might involve in the pathophysiology of MDD (Saleh et al., 2017; Bustamante et al., 2018; Osborne et al., 2018), while fatty acids (FAs) play an important role in the balance between the anti- and pro-inflammatory responses (Fritsche, 2015; Fraser et al., 2022), epigenetic regulation (Kulkarni et al., 2011; Lee et al., 2017), brain health (Rebello, 2022), and stress hormones (Nemeth et al., 2021). This provides some theoretical basis for the use of FA levels as one of the biological markers for the diagnosis of depression. Meanwhile, new evidence suggests that dietary intake of fish or alterations in peripheral FA composition have a role in the development of MDD (Marx et al., 2017; Cribb et al., 2018; Hamazaki et al., 2018; Scola et al., 2018) and influencing treatment response of mental disorders (de Almeida et al., 2020; Li et al., 2022). FAs and their metabolites are the key messengers of several important pathophysiological processes, including neurotransmission, neuroinflammation, and cell survival (Bazinet and Layé, 2014). Much attention has been devoted to the study of n-3 polyunsaturated fatty acids (PUFA) levels and n-3/n-6 PUFA ratio, which have been found to be lowered in serum/plasma and the red blood cell membrane of depressed patients (Edwards et al., 1998; Tiemeier et al., 2003; Lin et al., 2010). Antidepressant treatment and other antipsychotic medication might also influence FA metabolism; changes in the n-3 PUFAs level or n-3 index were reported in schizophrenia patients after antipsychotic treatment in previous research (Li et al., 2022). Metabolomics research has suggested that lipid membrane remodeling may play a role in selective serotonin reuptake inhibitor treatment response (MahmoudianDehkordi et al., 2021). Evidence from animal studies have also revealed that a decrease in PUFAs might explain the variation in individual response to fluoxetine (Tkachev et al., 2021). Furthermore, numerous clinical trials have demonstrated efficacy of certain n-3 PUFAs as a depression adjuvant therapy (Jazayeri et al., 2008; Gertsik et al., 2012; Jahangard et al., 2018), and changes in docosahexaenoic acid (DHA) levels after fish oil supplementation were associated with the improvement of clinical symptoms among depressed patients (Chhetry et al., 2016; Ganança et al., 2017). Thus, changes in DHA may be used as a potential marker for the treatment response in depression. Our previous research has found abnormal FA levels in depressed patients, although using FAs alone as the biological marker is insufficient to distinguish depression from general population. The published results indicated the high heterogeneity in depression and also inspired us to investigate if the change of FA metabolism represents a unique subgroup of depression with different clinical characteristics (Wang et al., 2022). Taken together, FA levels are potential biological indicators of depression or a subtype of depression, and alterations in FA metabolism might be associated with the treatment response for depression.

Human FAs are mainly synthesized in the liver from various precursors. Studies related to FA composition in humans have mostly used serum, plasma, or erythrocyte membranes (Lin et al., 2010; Watson et al., 2018), with only a few clinical studies extracting adipose tissue from patients (Mamalakis et al., 2006). Because erythrocytes cannot synthesize long-chain FAs de novo, their FA source is dependent on the Lands cycle (Lands, 1958). The primary enzymes involved are lysophospholipid acyltransferase and phospholipase A2, which alter cellular phospholipids through a series of deacylation and reacylation steps (Renooij et al., 1974). FAs can be taken up by erythrocyte membranes from the circulatory system, and the composition of erythrocyte membrane FAs can reflect the quantities of FAs in serum/plasma to some extent. Because erythrocyte membranes reflect FA metabolism over a longer length of time than serum/plasma (Carver et al., 2001; Assies et al., 2010) and are more accessible than adipose tissue, they have emerged as one of the most useful media for assessing FA metabolism in vivo.

The link between clinical characteristics of depression and membrane FA levels has yet to be established. The heterogeneity of published results was primarily caused by sex, age, diets, smoking, the use of antidepressants, and recurrent episode of disease. Furthermore, the role of n-6 PUFAs and other FAs (saturated FAs [SFAs], monounsaturated fatty acids [MUFAs], and trans FAs [TFAs]) in depression is not as conclusive as the involvement of n-3 PUFAs. Thus, many types of FAs were measured in red blood cells of patients with first-diagnosed, drug-naïve depression in the present study. The chance of taking antidepressants and recurrent courses of depression that would affect the FA metabolism were excluded. We aimed to explore the influence of depressive and anxious symptoms on the FA composition of erythrocyte membranes in MDD to determine if erythrocyte membrane FA levels could be used as a biological marker of depression.

## METHODS

### Participants

The cross-sectional study was conducted in the Second Xiangya Hospital of Central South University between June 2017 and January 2020. Participants aged 18–50 years with first-diagnosed, drug-naive depression were recruited. They were diagnosed by 2 experienced psychiatrists using the diagnostic criteria of the DSM-5. All patients who scored <21 on the 24-item Hamilton Depression Rating Scale (HAMD) were excluded, as were those who had any of the following conditions: (1) other serious illnesses or comorbidities, (2) other severe mental disorders (except for MDD), (3) apparent suicide attempt or behavior, (4) a history of psychoactive drug abuse (excluding alcohol and tobacco), (5) taking benzodiazepines daily, or (6) electroconvulsive therapy was conducted in the past 6 months or is required for the present episode. All of the healthy controls (HCs) who were recruited had no previous or current psychiatric diagnosis. Sex, age, and education level were similar between HC and depression groups. The exclusion and inclusion criteria of HCs were based on the previous study (Yang et al., 2022). Patients with psychiatric abnormalities in the HC group were excluded through Symptom Checklist-90 and psychiatric interviews conducted by trained interviewers. All participants were informed of the purposes, benefits and risks. Signed informed consents were also provided. The protocol of this study was reviewed and approved by the Ethics Committee of the Second Xiangya Hospital of Central South University (MDD201610).

### Clinical Assessment

Demographics and clinical characteristics were collected for each patient. HAMD was used to assess the depressive symptoms, and Hamilton Anxiety Scale (HAMA) was used to conduct assessments on anxiety symptoms. Patients were divided into severe depression (HAMD ≥ 35) and mild to moderate depression (21 ≤ HAMD < 35) according to the HAMD score. They were also divided into severe anxiety (HAMA ≥ 29) and mild to moderate anxiety (14 ≤ HAMD < 29) based on HAMA score. All participants completed the Beck Depression Inventory (BDI) and Self-Rating Anxiety Scale (SAS) assessments. The BDI and SAS are used to accurately reflect the subjective feelings of patients with depression and anxiety, respectively.

### Sample Preparation

Fasting blood samples were collected between 8:00 and 10:00 am. Then erythrocytes were separated and stored in a −80°C refrigerator until further analysis. The FA of erythrocyte membrane was extracted and purified using a previously reported method (Li et al., 2022).

### FA Composition Assays

The FAs of erythrocyte membrane were measured using an Agilent 7890A/5975C gas chromatograph-mass spectrometer, the details of which were referred to in a previous study (Li et al., 2022). The FA methyl esters were separated on a VF-23 ms column (Agilent Technologies, CA, USA): 30 m (length), I.D. 250-μm-wide bore, film thickness of 0.25 μm, and maximum operating temperature of 260°C.

The sum of n-3 PUFAs was defined as C20:5n3 (eicosapentaenoic acid [EPA]) + C22:5n3 (docosapentaenoic acid [DPA]) + DHA; the sum of n-6 PUFAs was defined as C18:2n6c (linoleic acid [LA]) + C20:3n6 (eicosatrienoic acid [EET]) + C20:4n6 (arachidonic acid [AA]) + C22:4n6 (docosatetraenoic acid [DTA]); and the sum of n-6 and n-3 PUFAs was total PUFAs. Desaturase activity is estimated using the ratio of FA product to precursor, called the desaturase index. The ratio of C20:3n6/C18:2n6 was used to estimate D6 desaturase activity and C20:4n6/C20:3n6 to estimate D5 desaturase activity. The n-6/n-3 ratio was used to assess the balance of n-6 PUFAs and n-3 PUFAs in the erythrocyte membrane. The elongation process for n-6 and n-3 was estimated using the ratios of C22:4n6/C20:4n6 and C22:5n3/C20:5n3, respectively.

### Statistical Analysis

Categorical data were analyzed by chi-square test or Fisher exact probability method. The Kolmogorov-Smirnov test was used to estimate the normality of measurement data. Normally distributed data were expressed as mean ± SD. The data with equal variance (tested by Levene test) was analyzed using 1-way ANOVA followed by a Tukey-Kramer test to compare the differences among the 3 groups; otherwise, it was analyzed by Welch ANOVA followed by Games-Howell test. Non-normally distributed data were analyzed by Kruskal-Wallis H test to compare the significant differences among the 3 groups with Bonferroni correction, which was expressed as interquartile range (quartile, P25–P75). Multiple comparisons were used in mild to moderate, severe depression or anxiety and HCs to clarify which 2 groups had significantly different levels of erythrocyte membrane FAs. The receiver operating characteristic (ROC) curve was used to identify potential biomarkers in distinguishing depression and anxiety severity. Area under the curve (AUC) values ≥0.7 were considered as potential biomarkers. All results were performed using IBM SPSS Statistics 22.0 and GraphPad Prism 8.0.

## RESULTS

### Demographic Data and Clinical Characteristics of Depressed Patients and HCs

Demographic data and clinical characteristics are listed in Tables 1 and 2. A total of 31 patients with severe depression, 108 patients with mild to moderate depression, and 55 HCs were included in the study. There were no statistically significant differences in age, gender, body mass index, education years, family history, and current smoking rate among the 3 groups, but there were significant differences in current drinking rate. Scale assessment results showed that the HAMD (*P* < .000) and HAMA scores (*P* < .000) in the severe depression group were significantly higher than those in the mild to moderate depression group. In addition, the BDI and SAS scores of the patients with severe depression were significantly higher than those of the HCs (*P* < .000; *P* < .000) and patients with mild to moderate depression (*P* = .001; *P* = .002). They were also higher in the mild to moderate depression compared with HCs (*P* < .000; *P* < .000).

**Table 1.** Demographic Data and Clinical Characteristics of Depression Patients and HCs

Characteristic	HCs (n = 55)	Mild to moderate depression (n = 108)	Severe depression (n = 31)	Statistics	*P* value
Age, mean (SD), y	29.1 (8.8)	27.7 (8.8)	27.7 (8.6)	0.606	.547
Female, n (%)	31 (56.4)	91 (65.7)	21 (67.7)	1.488	.475
BMI (kg/m^2^)	21.9 (2.7)	21.5 (3.0)	21.1 (2.5)	1.021	.363
Education year	14.1 (2.9)	13.9 (3.3)	13.5 (2.5)	0.375	.688
Family history, n (%)	5 (9.1)	22 (23.0)	9 (29.0)	5.964	.051
Current smoking, n (%)	7 (12,7)	15 (14.3)	5 (16.1)	0.287	.886
Current drinking, n (%)	0 (0)	3 (2.9)	4 (12.9)	7.500	.012*
HAMD	—	27.0 (4.5)	38.8 (4.0)	13.985	<.000**
HAMA	—	21.0 (6.2)	28.1 (5.2)	5.810	<.000**
BDI	5.5 (5.3) ^a,b^	27.0 (10.1)	33.5 (8.3) ^c^	121.2	<.000**
SAS	39.7 (7.7)^a,b^	54.0 (11.3)	60.6 (9.5) ^c^	62.64	<.000**

Abbreviations: BMI, body mass index; BDI, Beck Depression Inventory; HAMA, Hamilton Anxiety Scale; HAMD, 24-item Hamilton Depression Rating Scale; HC, healthy control; SAS, Self-Rating Anxiety Scale. *P < .05, **P < .01: significantly different among 3 groups.

^a^ *adjusted P* < .05: significantly different between moderate and severe depression and HC.

^b^ *adjusted P* < .05: significantly different between severe depression and HC.

^c^ *adjusted P* < .05: significantly different between mild to moderate and severe depression.

**Table 2.** Demographic Data and Clinical Characteristics of Anxiety Patients and HCs

Characteristic	HCs (n = 55)	Mild to moderate anxiety (n = 111)	Severe anxiety (n = 25)	Statistics	*P* value
Age, mean (SD)	29.1 (8.8)	27.4 (8.8)	29.1 (8.6)	0.917	.401
Female, n (%)	31 (56.4)	73 (65.8)	17 (68.0)	1.668	.464
BMI (kg/m^2^)	21.9 (2.7)	21.3 (2.8)	22.0 (3.1)	1.371	.257
Education year	14.1 (2.9)	13.7 (2.9)	14.2 (4.3)	0.395	.675
Family history, n (%)	5 (9.1)	26 (23.6)	5 (20.0)	5.290	.063
Current smoking, n (%)	7 (12,7)	17 (15.3)	3 (12.0)	0.238	.956
Current drinking, n (%)	0 (0)	5 (4.5)	2 (8.0)	3.842	.122
HAMD	—	28.4 (5.8)	35.9 (6.4)	−5.783	.000**
HAMA	—	20.5 (5.2)	32.3 (2.8)	−16.02	.000**
BDI	6.9 (7.8)^a^^,^^b^	27.2 (9.5)	34.8 (8.3)^c^	124.6	.000**
SAS	31.9 (6.1)^a^^,^^b^	43.4 (7.8)	50.8 (6.9)^c^	71.89	.000**

Abbreviations: BMI, body mass index; BDI, Beck Depression Inventory; HAMA, Hamilton Anxiety Scale; HAMD, 24-item Hamilton Depression Rating Scale; HC, healthy control; SAS, Self-Rating Anxiety Scale. *P* < .05, **P < .01: significantly different among 3 groups.

^a^ *adjusted P* < .05: significantly different between moderate and severe anxiety and HC.

^b^ *adjusted P* < .05: significantly different between severe anxiety and HC.

^c^ *adjusted P* < .05: significantly different between mild to moderate and severe anxiety.

According to the severity anxiety, patients were divided into severe anxiety (n = 25) and mild to moderate anxiety (n = 111). Age, gender, body mass index, education years, family history, current drinking and smoking rates were not significantly different among the HCs or mild to moderate and severe anxiety group. However, the severe anxiety patients had significantly higher HAMD scores (*P* < .000) and HAMA scores (*P* < .000) than the mild to moderate anxiety patients. There were significant differences in BDI and SAS scores among the 3 groups, and the BDI and SAS scores in the severe anxiety patients were significantly higher than those in the mild to moderate anxiety patients (*P* < .000; *P* < .000) and the HCs (*P* < .000; *P* < .000). This trend was also present in mild to moderate anxiety and HCs.

### Comparison of Erythrocyte Membrane FA Levels in Severe Depression and HCs

The FA levels of the patients with different severities of depression are listed in Table 3. Depressed patients had elevated levels of most FAs [besides C18:1n9c (oleic acid) compared with HCs. Specifically, the levels of C16:0 (palmitic acid; *P* = .028), C18:0 (stearic acid; *P* = .015), EA (*P* = .001), EET (*P* = .001), AA (*P* = .002), EPA (*P* = .021), DTA (*P* = .002), DPA (*P* = .004), DHA (*P* = .028), total FAs (*P* = .003), total SFAs (*P* = .016), total MUFAs (*P* = .002), total PUFAs (*P* = .001), n-6 (*P* = .002), and n-3 PUFAs (*P* = .010) in patients with severe depression were significantly increased compared with HCs, and the levels of stearic acid (*P* = .026), C18:1n9t (*P* = .009), EET (*P* = .023), AA (*P* = .005), EPA (*P* = .025), DTA (*P* = .002), DPA (*P* = .028), DHA (*P* = .034), total FAs (*P* = .012), total MUFAs (*P* = .006), total PUFAs (*P* = .006), n-6 (*P* = .009), and n-3 PUFAs (*P* = .031) were significantly elevated compared with patients with mild to moderate depression. Interestingly, the level of C18:1n9c decreased in depressed patients. Compared with HCs, C18:1n9c levels were significantly lower in patients with mild to moderate depression (*P* = .002).

**Table 3.** Fatty Acid Levels of Erythrocyte Membrane in Severity Depression and HCs

Fatty acid	HCs (n = 55)	Mild to moderate (n = 108)	Severe (n = 31)	Statistics	*P* value
C16:0	7.42 (4.98–12.72)^a^	9.01 (5.58–12.25)	11.63 (7.30–16.53)	6.809	.033*
C18:0	4.13 (2.75–9.02)^a^	4.93 (3.59–7.30)	8.24 (4.37–10.34)^c^	8.768	.012*
C18:1n9t	5.08 (2.71–7.51)^a^	5.65 (4.16–7.18)	8.42 (5.30–10.77)^c^	13.036	.001**
C18:1n9c	0.59 (0.16–0.59)^b^	0.25 (0.10–0.37)	0.28 (0.13–0.55)	11.964	.003**
C18:2n6c	3.40 (2.25–5.02)	3.85 (3.16–5.49)	3.95 (3.18–11.57)	4.654	.098
C20:3n6	0.24 (0.12)^a^	0.28 (0.12)	0.35 (0.14)^c^	7.256	.000**
C20:4n6 AA	3.41 (1.57–5.60)^a^	3.94 (2.10–5.52)	6.47 (4.17–8.16)^c^	12.599	.002**
C20:5n3 EPA	0.04 (0.02–0.08)^a^	0.04 (0.02–0.07)	0.07 (0.05–0.09)^c^	8.383	.015*
C22:4n6	0.53 (0.28–0.96)^a^	0.69 (0.32–0.91)	1.00 (0.83–1.30)^c^	13.326	.001**
C22:5n3	0.38 (0.17–0.72)^a^	0.51 (0.25–0.70)	0.67 (0.56–0.89)^c^	10.534	.005**
C22:6n3 DHA	0.75 (0.25–1.09)^a^	0.74 (0.36–1.14)	1.10 (0.71–1.40)^c^	7.749	.021*
Total FAs	25.31 (15.66–45.60)^a^	30.25 (21.83–40.59)	42.62 (28.76–58.17)^c^	11.701	.003**
Total SFAs	11.54 (7.28–21.70)^a^	14.18 (9.40–19.45)	18.69 (12.38–26.82)	8.028	.018*
Total MUFAs	5.16 (3.03–7.84)^a^	5.81 (4.39–7.40)	8.59 (5.45–11.39)^c^	12.540	.002**
Total PUFAs	8.83 (4.88–14.71)^a^	10.36 (6.99–13.72)	15.13 (10.26–22.33)^c^	13.016	.001**
n-6	7.45 (4.39–12.68)^a^	8.98 (6.33–11.83)	13.09 (8.93–19.52)^c^	12.520	.002**
n-3	1.21 (0.45–1.95)^a^	1.32 (0.69–1.83)	1.88 (1.32–2.34)^c^	9.109	.011*
n-6/n-3	8.25 (5.59–10.12)	7.69 (5.79–9.90)	7.09 (5.26–9.96)	0.122	.941
UI	1.30 (0.21)	1.33 (0.25)	1.39 (0.26)	1.521	.221
(EPA+DHA)/total FAs	0.02 (0.02–0.03)	0.02 (0.02–0.03)	0.03 (0.02–0.04)	0.650	.723
C18:0/C16:0	0.62 (0.49–0.71)	0.57 (0.49–0.66)	0.60 (0.53–0.82)	4.219	.121
C18:1n9/C18:0	1.05 (0.93–1.25)	1.10 (0.94–1.33)	1.20 (0.93–1.35)	3.429	.180
C20:3n6/C18:2n6	0.06 (0.03–0.11)	0.06 (0.04–0.10)	0.06 (0.03–0.11)	0.055	.973
C20:4n6/C20:3n6	16.96 (8.75)	15.43 (8.19)	18.59 (8.30)	0.493	.612
C22:4n6/C20:4n6	0.18 (0.15–0.20)	0.17 (0.14–0.19)	0.17 (0.15–0.20)	3.752	.153
C22:5n3/C20:5n3	8.28 (6.00–13.42)	10.26 (6.01–15.75)	10.18 (8.58–12.89)	1.989	.370

AA, arachidonic acid; DHA, docosahexaenoic acids; EPA, eicosapentaenoic acid; FA, fatty acid; HC, healthy control; MUFA, monounsaturated fatty acid; PUFA, polyunsaturated fatty acid; SFA, saturated fatty acid; UI, unsaturation index. *P < .05, **P < .01: significantly different among 3 groups. The data are expressed as (quartile, P25–P75) or mean ± SD.

^a^ *adjusted P* < .05: significantly different between severe depression and HC.

^b^ *adjusted P* < .05: significantly different between mild to moderate depression and HC .

^c^ *adjusted P* < .05: significantly different between mild to moderate and severe depression.

### Comparison of Erythrocyte Membrane FA Levels in Severe Anxiety and HCs

**Figure 1.**
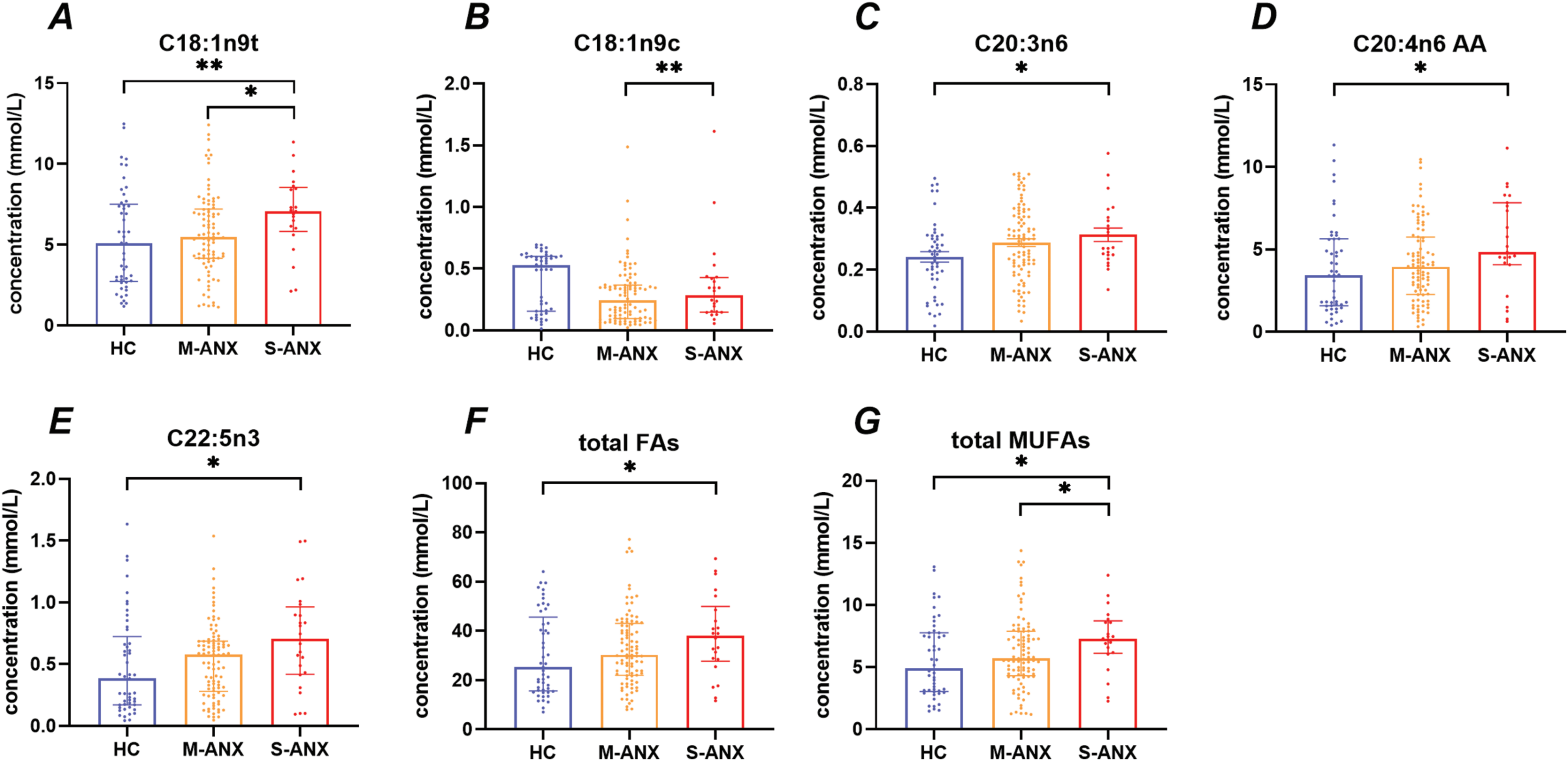
Fatty acid (FA) levels in severity anxiety and healthy controls (HCs). AA, arachidonic acid; HAMA, Hamilton Anxiety Scale; MUFA, monounsaturated fatty acid; M-ANX, mild to moderate anxiety; S-ANX, severe anxiety. *P < .05, **P < .01.

The FA levels of the patients with severity anxiety are shown in Table 4 and Figure 1. Except for C18:1n9c, the levels of most FAs were increased in the anxiety patients compared with HCs. Concretely, patients with severe anxiety had significantly higher levels of EA (*P* = .006), EET (*P* = .016), AA (*P* = .030), DPA (*P* = .028), total FAs (*P* = .043), and total MUFAs (*P* = .012) compared with HCs. Levels of EA (*P* = .044) and total MUFAs (*P* = .045) were significantly elevated in patients with severe anxiety compared with patients with mild to moderate anxiety. In addition, the level of oleic acid showed a downward trend in anxiety patients. Compared with HCs, C18:1n9c levels were significantly lower in patients with mild to moderate anxiety (*P* = .001).

**Table 4.** Fatty Acid Levels of Erythrocyte Membrane in Severity Anxiety and HCs

Fatty acid	HCs (n = 55)	Mild to moderate (n = 111)	Severe (n = 25)	Statistics	*P* value
C16:0	7.41 (4.98–12.72)	9.01 (5.85–12.84)	10.73 (7.22–15.68)	4.451	.108
C18:0	4.13 (2.75–9.02)	4.91 (3.66–7.61)	6.89 (4.31–10.06)	5.564	.062
C18:1n9t	5.08 (2.71–7.51)^a^	5.62 (4.17–7.53)	7.22 (6.05–9.39)^c^	9.602	.008**
C18:1n9c	0.53 (0.16–0.60)^b^	0.25 (0.10–0.37)	0.28 (0.15–0.43)	13.663	.001**
C18:2n6c	3.40 (2.25–5.02)	3.84 (3.20–5.49)	3.96 (2.92–10.39)	3.059	.217
C20:3n6	0.24 (0.12)^a^	0.29 (0.13)	0.33 (0.13)	4.534	.012*
C20:4n6 AA	3.43 (1.58–5.64)^a^	3.97 (2.29–5.94)	5.01 (4.10–8.16)	6.662	.036*
C20:5n3 EPA	0.04 (0.02–0.08)	0.05 (0.03–0.07)	0.07 (0.04–0.09)	5.043	.080
C22:4n6	0.53 (0.28–0.96)	0.71 (0.45–0.94)	0.97 (0.51–1.31)	5.003	.082
C22:5n3	0.38 (0.17–0.72)^a^	0.58 (0.28–0.69)	0.71 (0.42–0.96)	6.760	.034*
C22:6n3 DHA	0.75 (0.25–1.09)	0.79 (0.45–1.17)	0.98 (0.61–1.40)	2.646	.266
Total FAs	25.31 (15.66–45.60)^a^	30.66 (22.01–43.42)	39.00 (28.53–56.10)	6.019	.049*
Total SFAs	11.54 (7.28–21.71)	14.51 (9.49–19.88)	17.49 (11.21–25.24)	4.672	.097
Total MUFAs	5.16 (3.05–7.84)^a^	5.74 (4.35–7.92)	7.46 (6.27–9.93)^c^	8.562	.014*
Total PUFAs	8.83 (4.86–14.69)	10.81 (7.22–14.98)	12.38 (9.24–21.61)	5.677	.059
n-6	7.60 (4.38–13.01)	9.36 (6.68–12.33)	10.84 (7.50–17.93)	5.276	.071
n-3	1.21 (0.45–1.95)	1.36 (0.74–1.87)	1.76 (1.02–2.60)	4.297	.117
n-6/n-3	8.19 (5.59–10.12)	7.57 (5.71–9.84)	7.15 (5.15–11.12)	0.000	1.000
UI	1.30 (0.21)	1.34 (0.25)	1.35 (0.26)	0.734	.481
(EPA+DHA)/total FAs	0.02 (0.02–0.03)	0.03 (0.02–0.03)	0.03 (0.02–0.04)	0.056	.972
C18:0/C16:0	0.62 (0.49–0.71)	0.57 (0.50–0.66)	0.61 (0.53–0.74)	2.910	.233
C18:1n9/C18:0	1.05 (0.93–1.25)	1.13 (0.94–1.31)	1.15 (0.97–1.42)	3.385	.184
C20:3n6/C18:2n6	0.06 (0.03–0.09)	0.06 (0.03–0.10)	0.07 (0.04–0.13)	0.519	.771
C20:4n6/C20:3n6	16.96 (8.75)	16.56 (8.84)	18.06 (9.30)	0.277	.758
C22:4n6/C20:4n6	0.18 (0.15–0.20)	0.17 (0.14–0.19)	0.17 (0.14–0.19)	2.493	.287
C22:5n3/C20:5n3	8.28 (6.00–13.42)	10.32 (6.23–15.60)	9.66 (7.13–12.87)	1.898	.387

AA, arachidonic acid; DHA, docosahexaenoic acids; EPA, eicosapentaenoic acid; FA, fatty acid; HC, healthy control; MUFA, monounsaturated fatty acid; PUFA, polyunsaturated fatty acid; SFA, saturated fatty acid; UI, unsaturation index. *P < .05, **P < .01: significantly different among 3 groups. The data are expressed as (quartile, P25–P75) or mean ± SD.

^a^ *adjusted P* < .05: significantly different between severe anxiety and HC.

^b^ *adjusted P* < .05: significantly different between mild to moderate anxiety and HC.

^c^ *adjusted P* < .05: significantly different between mild to moderate and severe anxiety.

### ROC Analysis of FAs for Distinguishing Severity Depression

**Figure 2.**
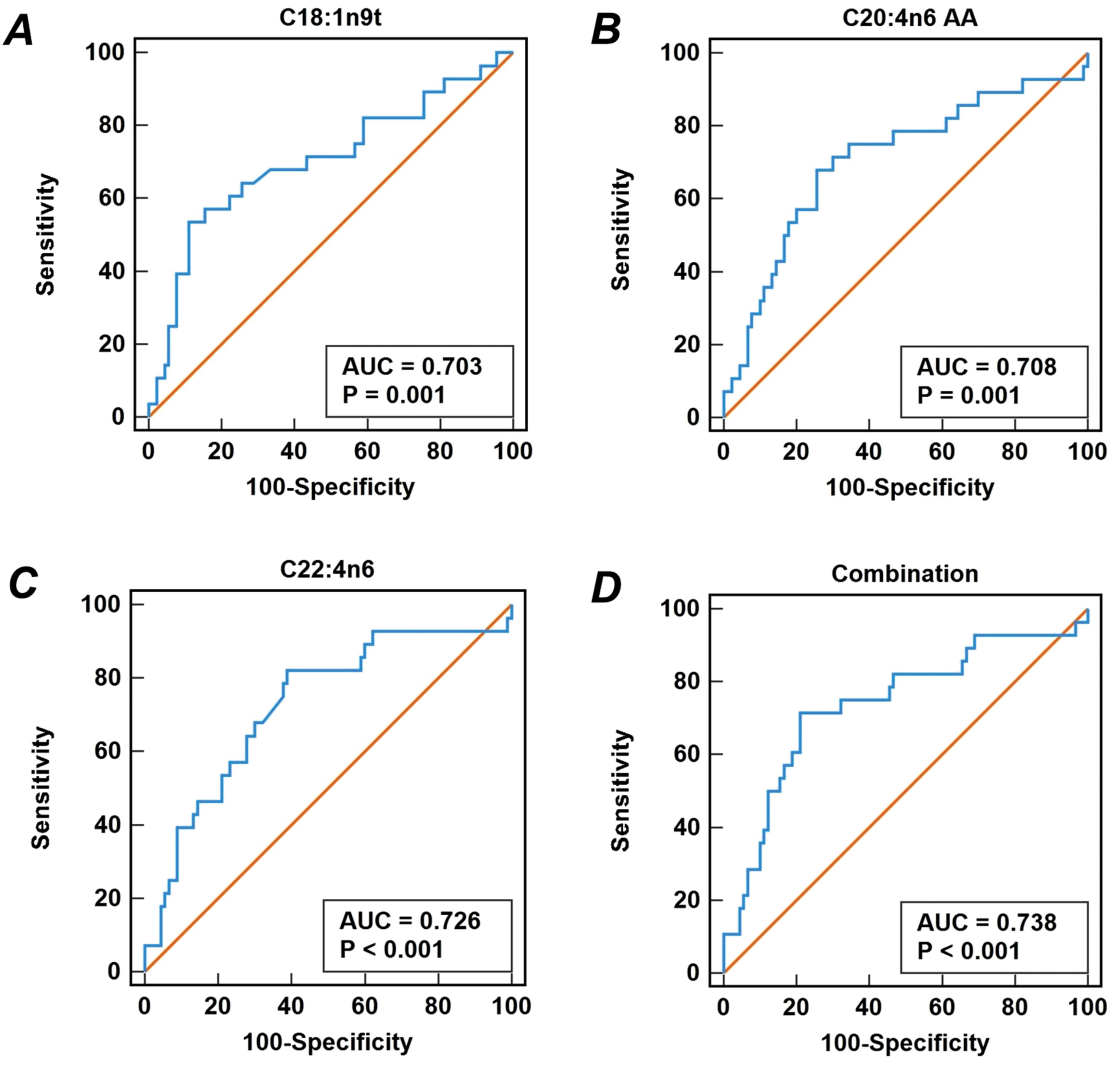
Receiver operating characteristic (ROC) curve of fatty acids for distinguishing severe depression. Combination is the ROC curve for the joint index of C18:1n9t, C20:4n6 AA, and C22:4n6. AA, arachidonic acid; AUC, area under the curve.

ROC analysis is shown in the Table 5 and Figure 2. Statistically significant FA indicators were used to differentiate between mild to moderate and severe depressed patients by ROC analysis. Indicators with area under the curve >0.7 were considered potential biomarkers of depression, including EA (area under the curve = 0.703; 95% confidence interval [CI] = 0.581–0.825), AA (area under the curve = 0.708; 95% CI = 0.589–0.826), and DTA (area under the curve = 0.726; 95% CI = 0.615–0.838). “Combination” was a new joint variable, which was established by EA, AA, and DTA using logistic regression model, and the area under the curve for distinguishing the severity depression was 0.738 (95% CI = 0.624–0.853). In addition, the results of FA indicators differentiating anxiety severity showed that the area under the curve without indicators was >0.7 (supplementary Table 1).

**Table 5.** ROC Analysis of Fatty Acids for Distinguishing Severe Depression

Parameter	AUC	*P* value	95% Cl	Cut-off	Sensitivity	Specificity
C18:1n9t	0.703 ± 0.062	.001	0.581–0.825	8.257	0.536	0.889
C20:4n6 AA	0.708 ± 0.061	.001	0.589–0.826	4.819	0.679	0.744
C22:4n6	0.726 ± 0.057	.000	0.615–0.838	0.796	0.821	0.611
Combination^a^	0.738 ± 0.058	.000	0.624–0.853	0.745	0.714	0.789

Abbreviations: AA, arachidonic acid; AUC, area under the curve; CI, confidence interval; ROC, receiver operating characteristic.

^a^Combination is the joint index of C18:1n9t, C20:4n6 AA and C22:4n6.

## DISCUSSION

This study investigated the relationship between erythrocyte FA levels and clinical symptoms in depression. The major findings are as follows: (1) levels of erythrocyte membrane FA in almost all kinds were elevated among severe depression compared to mild to moderate depression or compared to HCs (except for C18:1n9c); (2) total FAs, total MUFAs, EA, DPA, EET, and AA levels were elevated in depressed patients with severe anxiety compared to those mild/moderate anxiety or compared to HCs; (3) the levels of AA, DTA, EA, and the combination of all 3 have the potential to distinguish the severity of depressive symptoms in depression.

We observed elevated levels of almost all types of FAs (including SFAs, MUFAs, and PUFAs) in erythrocyte membranes in patients with severe depression. Assies et al. also found elevated plasma levels of SFAs, MUFAs, PUFAs, and total FAs in patients with recurrent depression compared with HCs, although erythrocyte levels were inconsistent (Assies et al., 2010), which is partially consistent with our findings. The increase in overall FA levels may be caused by increased intake or decreased oxidative catabolism. A large observational study (n = 69 843) found that patients with severe mental illness were more likely to have higher intakes of obesogenic nutrients and more inflammatory diets than the general population, such as more pro-inflammatory foods (carbohydrates and TFAs) instead of anti-inflammatory foods (coarse grains, vegetables, and n-3 PUFAs) (Firth et al., 2018). A prospective cohort study also found that ultra-processed foods and low activity levels were high risk factors for depression (Gómez-Donoso et al., 2020). The Western diet, which is high in processed foods with fat and sugar, is bad for both gut and brain by reducing gut microbiome diversity and increasing inflammatory levels and the risk of depression (Marx et al., 2021). The microbiota–gut–brain axis has been linked to depression. In addition, reactive oxygen species that could be induced by overconsumption of SFAs have the utmost importance in the progression of depression (Nakamura et al., 2019; Bhatt et al., 2020). Previous studies have shown that palmitic acid induced chronic low-level systemic inflammation, and oxidative stress may result in increased risk of depression. Furthermore, SFAs (especially C16:0) are able to activate inflammatory responses inside adipocytes and lead to the accumulation of reactive oxygen species (Davis et al., 2009) and pro-inflammatory factors (Shah et al., 2008). These alterations might affect cell membrane integrity, resulting in aberrant monoamine neurotransmitter trafficking in the brain (Su, 2009). In summary, abnormalities in FA metabolism resulting from changes in diet or catabolism are involved in the progress of depression.

This study also found that n-6 PUFAs, such as AA and DTA, and TFAs are much more powerful in distinguishing the severity of depressive symptoms among medication-free patients. The n-6 PUFAs were found to be positively associated with antenatal depressive symptoms (Wong et al., 2021), and TFAs also showed a strong association with depressive symptoms in US adults (Liu et al., 2019). However, it is still too early to conclude the alterations of FAs levels are trait, state, or endophenotype biomarkers for depression, and it is also difficult to prove whether it could be used as treatment biomarkers. Previous studies have found inflammatory, neurotrophic, metabolic, neurotransmitter, and neuroendocrine markers are highly promising candidates (Strawbridge et al., 2017). Translocation of Gsalpha from lipid rafts toward a more facile activation of adenylyl cyclase, which is a novel peripheral biomarker, was recently discovered (Targum et al., 2022). Whole-blood circFKBP8 and circMBNL1 were also found to be potential biomarkers for the diagnosis of MDD. This study proved the potential clinical value of n-6 PUFAs or TFAs for reflecting the severe of depressive symptom instead of the diagnosis of MDD. In the future, follow-up study is needed to test their possibility as the indicator of treatment response.

Before moving forward to clinical translation, some crucial aspects need to be considered. It is still not clear whether the FA composition of the erythrocyte membrane is pathophysiologically or mechanistically warranted as a biological marker of depression. Previous studies have found that FA metabolism was changed in depressed brain tissue. MaNamara et al. found reduced levels of DHA in the orbitofrontal cortex of depressed patients at autopsy, and this alteration was more pronounced in female patients (McNamara et al., 2007), which is consistent with the higher prevalence of depression in females than in males. Other studies showed inconsistent results. Recent autopsy studies have found no statistical differences in the levels of PUFAs in the corpus callosum (Hamazaki et al., 2017), prefrontal cortex (Hamazaki et al., 2015), amygdala (Hamazaki et al., 2012), and olfactory cortex (Hamazaki et al., 2013) in patients with depression or bipolar disorder. However, these autopsy studies included small sample sizes. All the evidence suggested that imbalance in FA metabolism may not present in the whole brain but in specific brain regions or some cells with important functions. For example, EPA is enriched in brain resident immune cells (microglia), although it would not accumulate in the whole brain (Cisbani et al., 2021). The FA composition plays an important role in functionality of different cells, so a better understanding of FA metabolism in the brain could identify new targets to improve the prognosis of depression.

The second question we need to answer is whether the changes in FA metabolism are consistent peripherally and centrally. Previous studies have found that the levels of PUFAs in peripheral erythrocyte membranes are positively correlated with the levels in brain gray matter, although it is not completely clear whether and to what extent changes in peripheral biological indicators can reflect pathological alterations in the CNS (Makrides et al., 1994). In addition, the activity of enzymes involved in FA metabolism in the brain is extremely low (Rapoport, 2013); as a result, it is hypothesized that the level of FAs in the brain is mainly dependent on the supply from the peripheral FA pool. The present study found that levels of all FAs in erythrocyte membranes were elevated in patients with MDD, including the neuroprotective n-3 PUFAs. The results of the present study are contradictory to most studies if the levels of peripheral FAs are reflective of intracerebral levels. Several possible explanations are as follows. Firstly, the metabolic process of FAs is complex, including a wide range of linked enzymes and active chemicals. As a result, the amounts of FAs in different tissues may be difficult to explain using a simple linear relationship, and genetic factor, age, gender, and oxidative stress generated by lifestyle are also involved (Appleton et al., 2008; Hodson et al., 2008). Furthermore, our research focused on absolute FA levels rather than relative levels, making it harder to extrapolate results from 1 study to the next, whereas most previous related research has focused on relative levels. In summary, imbalances in FA metabolism in the brain may be only a characteristic alteration of a certain subtype of depression, and the nature of these FA alterations still needs to be elucidated in depression.

Currently, the diagnosis of psychiatric disorders relies on the physician’s assessment of symptoms using relevant scales. This study found that peripheral erythrocyte membrane FA levels have the potential to be the biomarker of patients with MDD. Despite the data pointing to a connection between depressive symptoms and an increase in FA levels, this should not be interpreted as indicating a deterministic relationship because there are several factors may confound or moderate this association. We cannot ignore the role of genetic inheritance, age, sex, and diet even though the use of antidepressants and recurrent depressive episodes have already been taken into account. Furthermore, a 6-year longitudinal study could not confirm uni- or bidirectional association n-3 PUFA plasma levels and depressive disorders and severity over time (Thesing et al., 2020). As a result, more work needs to be done to reveal the above issues.

Several limitations also need to be acknowledged. A major limitation is that some confounders that may potentially influence FA levels are not included in this study, such as activity, diet, and genetics. Although this study reported significant diagnosed effects of some FA levels in differentiating the severe depression from the depression group, this study was limited by the clinical translation for lack of mechanism research. Additionally, this study is only a cross-sectional exploratory investigation, so a causal relationship cannot be concluded, which would further limit the clinical translation. Furthermore, because of the limited variety of FA indicators tested in this paper, false discovery rate correction was not used. The imbalance in sample size due to the definition of depression severity may affect the statistical efficacy of this paper. Finally, only the erythrocyte FA ratios were used to estimate the activity of related enzymes; future studies detecting protease activity are warranted. The FA metabolism in different tissues is worth exploring as a result of the tissue variability in FA distribution and metabolism.

## CONCLUSION

The results suggest that erythrocyte membrane FA levels have the potential to be a biological marker in depression. However, the use of erythrocyte membranes alone as a diagnostic marker for depression is far from sufficient. It is foreseeable that future biological or clinical indicators for the diagnosis of depression must be multidimensional, and the findings of the present study lay a foundation for exploring the role of FA metabolism in the diagnosis of depression. In the future, more basic studies should examine the molecular mechanisms underpinning depressive symptoms and identify relevant biomarker changes. In addition, large prospective cohort studies and related clinical trials are needed to explore the causal association between FA metabolism and depression as well as the impact of erythrocyte FA metabolism on depression prevention, diagnose, treatment, and prognosis. By understanding all of these, we will be able to improve outcomes for depression.

## Supplementary Material

pyad021_suppl_Supplementary_Table

## Data Availability

The data that support the findings of this study are available from the corresponding author upon reasonable request.
