# Supplementary material for: Erythrocyte Membrane Fatty Acid Composition as a Potential Biomarker for Depression
Source: Int J Neuropsychopharmacol. 2023 May 22;26(6):385–95. doi: 10.1093/ijnp/pyad021 (PMC10289140; doi:10.1093/ijnp/pyad021)
Supplement: pyad021_suppl_Supplementary_Table [file pyad021_suppl_supplementary_table.docx]

Supplementary table S1. ROC analysis of fatty acids for differentiating severe anxiety

| Parameter | AUC | P-value | 95% Cl | Cut-off | Sensitivity | Specificity |
| --- | --- | --- | --- | --- | --- | --- |
| C16:0 | 0.594 ± 0.066 | 0.154 | 0.466-0.723 | 8.891 | 0.500 | 0.708 |
| C18:0 | 0.643 ± 0.068 | 0.031 | 0.509-0.777 | 5.241 | 0.596 | 0.708 |
| C18:1n9t | 0.674 ± 0.061 | 0.009 | 0.554-0.795 | 5.883 | 0.543 | 0.792 |
| C18:1n9c | 0.580 ± 0.061 | 0.230 | 0.460-0.699 | 0.125 | 0.330 | 0.917 |
| C18:2n6c | 0.505 ± 0.077 | 0.936 | 0.354-0.656 | 11.45 | 0.979 | 0.250 |
| C20:3n6 | 0.569 ± 0.060 | 0.297 | 0.451-0.687 | 0.248 | 0.404 | 0.792 |
| C20:4n6AA | 0.641 ± 0.068 | 0.033 | 0.508-0.775 | 4.060 | 0.532 | 0.792 |
| C20:5n3EPA | 0.637 ± 0.065 | 0.038 | 0.511-0.764 | 0.066 | 0.660 | 0.625 |
| C22:4n6 | 0.623 ± 0.072 | 0.065 | 0.481-0.764 | 1.207 | 0.915 | 0.417 |
| C22:5n3 | 0.631 ± 0.070 | 0.049 | 0.493-0.768 | 0.738 | 0.809 | 0.500 |
| C22:6n3DHA | 0.583 ± 0.068 | 0.209 | 0.450-0.717 | 1.217 | 0.787 | 0.417 |
| Total FA | 0.623 ± 0.067 | 0.063 | 0.492-0.754 | 32.66 | 0.553 | 0.667 |
| Total SFA | 0.615 ± 0.067 | 0.083 | 0.484-0.745 | 17.24 | 0.670 | 0.583 |
| Total MUFA | 0.674 ± 0.062 | 0.009 | 0.553-0.795 | 6.020 | 0.543 | 0.833 |
| Total PUFA | 0.609 ± 0.071 | 0.100 | 0.470-0.748 | 15.58 | 0.798 | 0.458 |
| n-6 | 0.597 ± 0.071 | 0.145 | 0.458-0.735 | 13.09 | 0.777 | 0.458 |
| n-3 | 0.598 ± 0.068 | 0.138 | 0.465-0.732 | 1.861 | 0.755 | 0.500 |
| n-6/n-3 | 0.504 ± 0.073 | 0.947 | 0.361-0.648 | 6.580 | 0.500 | 0.681 |
| UI | 0.531 ± 0.069 | 0.635 | 0.397-0.666 | 1.444 | 0.649 | 0.500 |
| (EPA+DHA)/total FA | 0.510 ± 0.071 | 0.878 | 0.372-0.649 | 0.017 | 0.333 | 0.777 |
| C18:0/C16:0 | 0.600 ± 0.062 | 0.132 | 0.478-0.721 | 0.577 | 0.625 | 0.602 |
| C18:1n9/C18:0 | 0.560 ± 0.069 | 0.367 | 0.424-0.696 | 1.381 | 0.333 | 0.830 |
| C20:3n6/C18:2n6 | 0.544 ± 0.070 | 0.504 | 0.408-0.681 | 0.138 | 0.250 | 0.936 |
| C20:4n6/C20:3n6 | 0.555 ± 0.066 | 0.411 | 0.424-0.685 | 14.27 | 0.750 | 0.394 |
| C22:4n6/C20:4n6 | 0.517 ± 0.063 | 0.799 | 0.393-0.640 | 0.218 | 0.958 | 0.160 |
| C22:5n3/C20:5n3 | 0.531 ± 0.057 | 0.640 | 0.418-0.644 | 13.21 | 0.833 | 0.362 |

Supplementary table S2. ROC analysis of fatty acids for differentiating between depression

| Parameter | AUC | P-value | 95% Cl | Cut-off | Sensitivity | Specificity |
| --- | --- | --- | --- | --- | --- | --- |
| C18:1n9t | 0.600 ± 0.051 | 0.050 | 0.522-0.674 | 3.684 | 0.826 | 0.449 |
| C18:1n9c | 0.662 ± 0.051 | 0.001 | 0.585-0.732 | 0.457 | 0.835 | 0.612 |
| C20:3n6 | 0.626 ± 0.047 | 0.008 | 0.549-0.699 | 0.321 | 0.405 | 0.837 |
| C20:4n6AA | 0.583 ± 0.050 | 0.098 | 0.505-0.658 | 1.827 | 0.818 | 0.408 |
| Total FAs | 0.590 ± 0.052 | 0.082 | 0.512-0.665 | 21.26 | 0.793 | 0.449 |
| n-6 PUFAs | 0.593 ± 0.052 | 0.070 | 0.826-0.429 | 6.006 | 0.826 | 0.429 |

Supplementary table S3. Chemical formula and English name of fatty acids

| Formula | Name | Abbreviation |
| --- | --- | --- |
| C16:0 | palmitic acid | PA |
| C18:0 | stearic acid | SA |
| C18:1n9t | elaidic acid | EA |
| C18:1n9c | oleic acid | OA |
| C18:2n6c | linoleic acid | LA |
| C20:3n6 | eicosatrienoic acid | EET |
| C20:4n6 | arachidonic acid | AA |
| C20:5n3 | eicosapentaenoic acid | EPA |
| C22:4n6 | docosatetraenoic acid | DTA |
| C22:5n3 | docosapentaenoic acid | DPA |
| C22:6n3 | docosahexaenoic acid | DHA |
